# Adaptive foveated single-pixel imaging with dynamic supersampling

**DOI:** 10.1126/sciadv.1601782

**Published:** 2017-04-21

**Authors:** David B. Phillips, Ming-Jie Sun, Jonathan M. Taylor, Matthew P. Edgar, Stephen M. Barnett, Graham M. Gibson, Miles J. Padgett

**Affiliations:** 1 School of Physics and Astronomy, University of Glasgow, Glasgow G12 8QQ, UK.; 2 Department of Opto-Electronic Engineering, Beihang University, Beijing 100191, China.

**Keywords:** foveated imaging, single-pixel imaging, computational imaging, compressive sensing, biomimetic technology, adaptive imaging

## Abstract

In contrast to conventional multipixel cameras, single-pixel cameras capture images using a single detector that measures the correlations between the scene and a set of patterns. However, these systems typically exhibit low frame rates, because to fully sample a scene in this way requires at least the same number of correlation measurements as the number of pixels in the reconstructed image. To mitigate this, a range of compressive sensing techniques have been developed which use a priori knowledge to reconstruct images from an undersampled measurement set. Here, we take a different approach and adopt a strategy inspired by the foveated vision found in the animal kingdom—a framework that exploits the spatiotemporal redundancy of many dynamic scenes. In our system, a high-resolution foveal region tracks motion within the scene, yet unlike a simple zoom, every frame delivers new spatial information from across the entire field of view. This strategy rapidly records the detail of quickly changing features in the scene while simultaneously accumulating detail of more slowly evolving regions over several consecutive frames. This architecture provides video streams in which both the resolution and exposure time spatially vary and adapt dynamically in response to the evolution of the scene. The degree of local frame rate enhancement is scene-dependent, but here, we demonstrate a factor of 4, thereby helping to mitigate one of the main drawbacks of single-pixel imaging techniques. The methods described here complement existing compressive sensing approaches and may be applied to enhance computational imagers that rely on sequential correlation measurements.

## Teaser

The performance of a single-pixel computational video camera is enhanced by mimicking the compressive features of animal vision.

## INTRODUCTION

Computational imaging encompasses techniques that image using single-pixel detectors in place of conventional multipixel image sensors (*1*, *2*). This is achieved by encoding spatial information in the temporal dimension (*3*). Using this strategy, images are reconstructed from a set of sequential measurements, each of which probes a different subset of the spatial information in the scene. This enables imaging in a variety of situations that are challenging or impossible with multipixel image sensors (*4*). Examples include imaging at wavelengths where multipixel image sensors are unavailable, such as in the terahertz band (*5*–*7*), three-dimensional (3D) ranging (*8*–*11*), and fluorescence imaging through precharacterized multimode fibers and scattering media (*12*–*15*).

To fully sample an unknown scene to a particular resolution, the minimum number of measurements required is equal to the total number of pixels in the reconstructed image. Therefore, doubling the linear resolution increases the required number of measurements by a factor of 4, leading to a corresponding reduction in frame rate. This trade-off between resolution and frame rate has led to the development of a range of compressive techniques that aim to use additional prior knowledge or assumptions about a scene to reconstruct images from an undersampled set of measurements (*16*–*20*).

Despite these challenges, computational imaging approaches also potentially offer new and more flexible imaging modalities. For example, the lack of a fixed Cartesian pixel geometry means that it is no longer necessary for the resolution or exposure time (that is, the time taken to record all the measurements used in the reconstruction of an image) to remain uniform across the field of view or constant from frame to frame (*21*–*24*).

A variety of animal vision systems successfully use spatially variant resolution imaging (*25*). For example, the retina in the vertebrate eye has a region of high visual acuity (the fovea centralis) surrounded by an area of lower resolution (peripheral vision) (*26*). The key to the widespread success of this form of foveated vision is in its adaptive nature. Our gaze, which defines the part of the scene that is viewed in high resolution during a period of fixation, is quickly redirected (in a movement known as a saccade) toward objects of interest (*27*, *28*). Unlike a simple zoom, the entire field of view is continuously monitored, enabling saccadic movement to be triggered by peripheral stimuli such as motion or pattern recognition (*29*–*31*). Space-variant vision exploits the temporal redundancy present in many dynamic scenes to reduce the amount of information that must be recorded and processed per frame, essentially performing intelligent lossy compression at the point of data acquisition. This, in turn, speeds up the frame rate of such a vision system and enables us to react to our surroundings more fluidly.

Here, we demonstrate how an adaptive foveated imaging approach can enhance the useful data gathering capacity of a single-pixel computational imaging system. We note that there has already been much interest in mimicking animal imaging systems for image compression and robotic vision (*32*–*34*), and our work extends this to the constricted bandwidth regimes of single-pixel computational imagers. We reduce the number of pixels in each raw frame (thereby increasing the frame rate) by radially increasing the size of pixels away from a high-resolution foveal region (*35*, *36*). The position of the fovea within the field of view can then be guided by a variety of different visual stimuli detected in previous images (*37*).

Furthermore, we also borrow a concept from the compound eye architecture to increase the resolution of our images in the periphery: the fusion of multiple low-resolution frames to synthesize a higher-resolution image of the scene [a technique also known as supersampling or digital superresolution (*38*–*40*)]. In this way, we rapidly record the details of fast-changing or important features in a single frame while simultaneously building up detail of more slowly changing regions over several consecutive frames. We show how this tiered form of digital superresolution enables the reconstruction of composite images that have both a spatially varying resolution and a spatially varying effective exposure time, which can be optimized to suit the spatiotemporal properties of the scene. We demonstrate an implementation of our technique with a single-pixel camera; however, the method can be applied to enhance the performance of a growing range of computational imaging systems that reconstruct images from a set of sequential correlation measurements.

## RESULTS

### Foveated single-pixel imaging

Single-pixel imaging is based on the measurement of the level of correlation between the scene and a series of patterns. The patterns can either be projected onto the scene [known as structured illumination (*1*); closely related to the field of computational ghost imaging (*41*–*43*)] or be used to passively mask an image of the scene, a technique known as structured detection (*2*), and the method we use here.

**Fig. 1:**
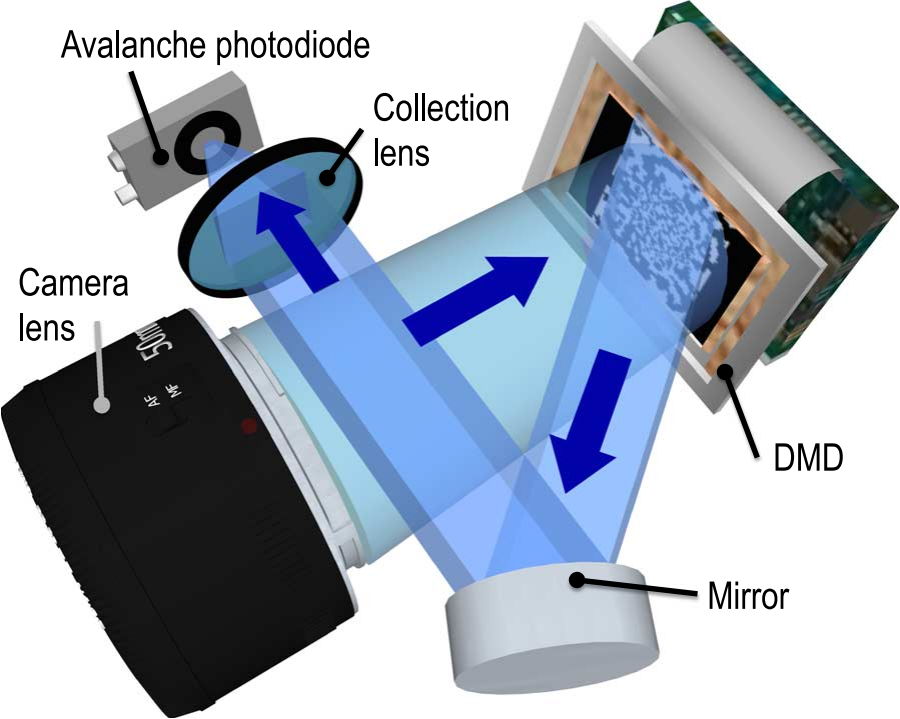
Experimental setup. The scene is flood-illuminated and imaged onto a DMD, which operates as a dynamic mask: Light from a subset of the micromirrors is reflected to an avalanche photodiode (APD), which records the total intensity transmitted by each binary masking pattern. More details are given in Materials and Methods.

A schematic of the single-pixel camera used in this work is shown in Fig. 1 based on the design previously demonstrated by Edgar *et al*. (*44*) and Sun *et al*. (*45*). A digital micromirror device (DMD) is placed at the image plane of a camera lens, that is, at the same plane where a multipixel camera sensor would be placed in a conventional camera. The DMD is used to rapidly mask the image of the scene with a set of binary patterns, and the total amount of light transmitted by each mask is recorded by a photodiode, representing a measurement of the level of correlation of each mask with the scene. Knowledge of the transmitted intensities and the corresponding masks enables reconstruction of the image.

By choosing a set of linearly independent masks, the scene can be critically sampled in an efficient manner using the same number of masks as the number of pixels in the reconstructed image. A mask set that is widely used for single-pixel imaging is formed from the Hadamard basis, which is a set of orthonormal binary functions with elements that take the value of +1 or −1 (*46*–*48*). This represents a convenient choice of expansion because, when represented on a DMD, each mask transmits light from approximately half of the image pixels, thus maximizing the signal at the photodiode.

**Fig. 2:**
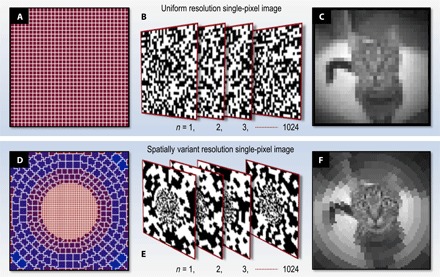
Single-pixel imaging with spatially variant resolution. (**A** to **C**) Uniform resolution. (A) Uniform 32 × 32–pixel grid with *N* = 1024 pixels. (B) Examples of a complete 1024 Hadamard pattern set (negatives not shown) reformatted onto the 2D uniform grid shown in (A). (C) Image of a cat recorded experimentally in ~0.125 s, reconstructed from the level of correlation with each of the 1024 masks shown in (B). (**D** to **F**) Spatially variant resolution. (D) Spatially variant pixel grid, also containing *N* = 1024 pixels of varying area. Within the fovea, the pixels follow a Cartesian grid, chosen to avoid aliasing with the underlying Cartesian grid of the DMD at high resolutions. Surrounding the fovea is a peripheral cylindrical polar system of pixels. (E) Examples of the 1024 Hadamard patterns reformatted onto the spatially variant grid shown in (A). (F) Image of the identical scene to that shown in (C), reconstructed here from correlations with the 1024 spatially variant masks shown in (E). In the central region of (F), the linear resolution is twice that of the uniform image (C).

A uniform-resolution *N* pixel image of the scene (represented here by an *N* element column vector **o**_*un*_) can be expressed as a linear sum of *N* Hadamard basis vectors, index *n* of which is denoted by **h**_*n*_

$$o_{un}=\frac{1}{N}\sum_{n=1}^{N}a_{n}h_{n}$$

where *a*_*n*_ is the level of correlation between the scene **o**_*un*_ (sampled to the same resolution as the Hadamard patterns) and mask *n* recorded by the photodiode; that is, *a*_*n*_ is measured by projecting the scene onto the *n*th Hadamard mask, *a*_*n*_ *=* **h**_*n*_^*T*^**o**_*un*_, which follows from the orthogonality of the Hadamard vectors (**h**_*n*_^*T*^**h**_*m*_ *= N*δ_*nm*_). We emphasize that any spatial frequency components in the scene that are above the spatial frequency limit of the uniform pixel grid are lost in this process. Although presented here in 1D vector notation, experimentally, a 2D image is recorded, with each 1D vector **h**_*n*_ being reshaped onto a uniform 2D grid that is displayed on the DMD as shown in Fig. 2 (A and B). More detail is given in Materials and Methods.

Figure 2C shows an example of an experimentally reconstructed image of uniform resolution containing 32 × 32 pixels. Exploiting a fast DMD (see caption of Fig. 1) enables the display of ~2 × 10^4^ masks/s, resulting in a reconstructed frame rate of ~10 Hz at this 32 × 32–pixel resolution [incorporating two patterns per pixel for differential measurement to improve signal-to-noise ratio (SNR); see sections S1 and S3]. Evidently, it is highly desirable to try to increase the useful resolution–frame rate product of such a single-pixel computational imaging system.

We use a modification of the technique described above to measure and reconstruct images of nonuniform resolution. In this case, the masking patterns displayed on the DMD are created by reformatting each row of the Hadamard matrix into a 2D grid of spatially variant pixel size, as shown in Fig. 2 (D and E). For clarity, we henceforth refer to these nonuniformly sized pixels as cells. Here, the 2D grid has an underlying Cartesian resolution of *M* = 64 × 64 = 4096 pixels but contains *N* = 1024 independent cells. Mathematically, this reformatting operation may be expressed as a transformation of the Hadamard basis vectors to a new set of vectors **s** using the matrix **T**, which is a *M* × *N* (rows × columns) binary matrix that stretches a vector of *N* elements (representing the number of cells) to populate a (larger) vector of *M* high-resolution pixels, **s**_*n*_ *=* **Th**_*n*_. Similarly to above, we measure the correlation *b*_*n*_ between each pattern **s**_*n*_ and the scene **o** (where, here, **o** is an *M* element vector representing the scene at uniform resolution equivalent to the highest resolution of patterns **s**). Therefore, *b*_*n*_ = **s**_*n*_^*T*^**o**.

Because of the stretch transformation, the masks **s** are no longer orthogonal. However, the spatially variant image of the scene, **o**_*sv*_, can still be efficiently reconstructed using

$$o_{sv}=A^{-1}\frac{1}{N}\sum_{n=1}^{N}b_{n}s_{n}$$

Here, **A** is an *M* × *M* diagonal matrix encoding the area of each pixel in the stretched basis: Element *A*_*mm*_ is equal to the area of the cell to which high-resolution pixel *m* belongs. Section S2 gives a detailed derivation of this result. Unlike before, in this case, the high spatial frequency cutoff is now spatially variant across the field of view. We also note that the SNR is now also spatially variant. See section S3 for more detail and for the definition of SNR used here.

Figure 2F shows an experimentally reconstructed, spatially variant resolution image of the same scene as shown in Fig. 2C. Although both of the images use the same measurement resource (that is, each has the same total number of independent cells, and therefore, each requires the same number of mask patterns and effective exposure time to measure), the linear resolution in the central region of Fig. 2F is twice that of Fig. 2C. The detail in the foveal region (the cat’s face) is therefore enhanced in Fig. 2F at the expense of lower resolution in the surroundings.

### Spatially variant digital supersampling

If the positions of the pixel boundaries are modified from one frame to the next, then each frame samples a different subset of the spatial information in the scene. Consequently, successive frames are capturing not only information about the temporal variation of the scene but also additional complementary information about the spatial structure of the scene. Therefore, if we know that a local region of the scene has been static during the course of the measurements, we can combine these measurements to recover an image of enhanced resolution compared to the reconstruction of an individual frame in isolation. This technique is known as digital superresolution or supersampling (*39*). Because the pixel geometry of each frame in our single-pixel imaging system is defined by the masking patterns applied to the DMD and used to measure the image, it is straightforward to modify the pixel boundaries from frame to frame as required, and the images are inherently co-registered for digital resolution enhancement. We note that the term digital superresolution refers to increasing the resolution in imaging systems in which the resolution is limited by the pixel pitch and not by the diffraction limit.

**Fig. 3:**
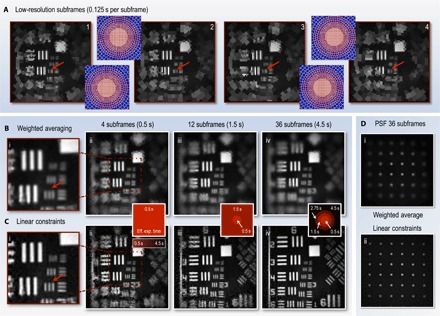
Reconstructing images with a spatially variant effective exposure time using digital supersampling. All images are reconstructed from experimental data. (**A**) Four subframes, each with the foveal cells shifted by half a cell in *x* and/or *y* with respect to one another (*45*). The number of cells in each subframe is *N* = 1024. The purple insets show the underlying cell grid in each case. Movie S1 shows the changing footprints of the subframes in real time (see section S7 for full description of movie S1). (**B**) Composite images reconstructed from increasing numbers of subframes using the weighted averaging method. (**C**) Composite images reconstructed from increasing numbers of subframes using the linear constraint method. The number of hr-pixels in the high-resolution composite images is *M* = 128 × 128 = 16,384, although not all of these may be independently recovered, depending on the number of sub-images combined and the configuration of each sub-image’s cells. Insets bridging (B) and (C) color-code the local time taken to perform the measurements used to reconstruct each region within the field of view, that is, the spatially variant effective exposure time of the images. The central region only uses data from the most recent four subframes (taking 0.5 s), whereas the reconstruction of the periphery uses data from subframes going progressively further back in time. Movie S2 shows a movie of the progressive linear constraint reconstruction (see section S7 for full description of movie S2). (**D**) Reconstructions of a uniform grid of points from 36 subframes to compare the PSF of the two reconstruction methods.

Figure 3 demonstrates how digital supersampling can be combined with spatially variant resolution, which leads to reconstructions with different effective exposure times across the field of view. For clarity, we henceforth refer to the raw images (shown in Fig. 3A) as subframes (which contain the nonuniformly sized cells) and the underlying uniform Cartesian pixels of the high-resolution composite reconstruction as hr-pixels. Our DMD can be preloaded with a set of masks to measure up to ~36 different subframes, each containing 1024 cells with different footprints, which, once loaded, can be played consecutively in an arbitrary and rapidly switchable order.

Within the fovea where the cells occupy a regular square grid, the linear resolution can be doubled by combining four subframes with overlapping fovea positions. To achieve this, we translated the cell footprints by half a cell’s width in the *x* and/or *y* direction with respect to the other subframes [more detail of the relative cell positions is given by Sun *et al*. (*45*)]. Movie S1 shows the recording of the subframes in real time (see section S7 for full description). The variation in detail within the fovea of each of the four subframes can be seen in Fig. 3A, where the red arrows highlight regions for comparison. The boundaries of the lower-resolution peripheral cells are also repositioned in different subframes in Fig. 3A, but because the peripheral cells form an irregular grid with variable sizes, they cannot be shifted by a constant amount. Instead, they are randomly repositioned with each new subframe, which is realized by randomizing their azimuth and displacing the center of each fovea by a small amount. Therefore, the resolution is increased nonuniformly in the periphery.

Having acquired this information, we are free to choose different reconstruction algorithms to fuse the information from multiple subframes to recover an improved estimate of the original scene **o**′_*sv*_. Here, we demonstrate and compare two such fusion strategies: weighted averaging and linear constraints. These reconstruction strategies are built upon different assumptions about the level of motion within the scene and trade off real-time performance against the quality of superresolution reconstruction. We note that, beyond the reconstruction techniques described here, more sophisticated approaches are possible, which, following compressive sensing techniques, can also incorporate additional priors relating to the structure of the scene. See section S6 for further discussion.

***Weighted averaging***

In this first strategy, we perform a weighted average of multiple subframes to reconstruct an image with increased resolution (*45*). The subframes are upscaled by a factor of 2 and are co-registered. Within the fovea, the four most recent subframes (Fig. 3A) are averaged with equal weightings, yielding a higher-resolution composite image as shown in Fig. 3B. Outside the foveal region, the sizes of the cells vary, and we choose weighting factors for each subframe that are inversely proportional to the area of the corresponding subframe cell where the data are taken from, promoting data from cells that have a smaller area and thus a higher local resolution. Weighting in this way incorporates local data from all subframes in every composite image hr-pixel, which has the benefit of suppressing noise. Alternate weighting factors are also possible; see section S4 for further discussion.

***Linear constraints***

Our second reconstruction strategy makes use of all available data in the measurements. The reconstructed intensity value of a single subframe cell represents an algebraic constraint on the sum of the group of hr-pixels that are members of that cell. Successive subframes are acquired with variations on the cell boundaries, which changes the group of hr-pixels corresponding to each subframe cell. As long as a local region of the image has remained static during acquisition of multiple subframes, these constraints can then be combined together into one self-consistent system of linear equations, which we solve using a constrained lowest mean square error (LMSE) technique to recover an improved estimate for the intensity value of each hr-pixel in the composite reconstruction, **o**′_*sv*_. Our constrained LMSE technique is suitable for systems that may be locally overdetermined, critically determined, or underdetermined, depending on the number of subframes available for the reconstruction. See section S5 for more details.

Movie S2 and Fig. 3 (B and C) compare reconstructions using these two alternative methods, as increasing numbers of subframes are used (see section S7 for full description of movie S2). For weighted averaging, the foveal region reaches a maximum resolution upon combination of the four most recent subframes with overlapping fovea (Fig. 3B, i), and further increasing the number of subframes averaged in the periphery smooths the reconstruction but does little to improve its resolution (Fig. 3B, ii to iv). With the linear constraint method, the maximum resolution in the foveal region is also reached after only four subframes (Fig. 3C, i). However, here, the point spread function (PSF) is sharper, and hence, high spatial frequencies are reproduced more faithfully. Furthermore, in the peripheral region, as larger numbers of subframes are fused into the reconstruction, the resolution continues to improve (Fig. 3C, ii to iv). Sun *et al*. (*45*) describe the reasons underpinning the lower resolution observed with the weighted mean reconstruction.

Thus, our tiered imaging system captures the detail of the central region of the scene at a frame rate of 8 Hz, with resolution-doubled images simultaneously delivered at a frame rate of 2 Hz. The weighted average method offers the same frame rates in the periphery, but with a space-variant broadening of the PSF, and hence reduced resolution (Fig. 3D, i). For static regions of the scene, the linear constraint method offers a means to further trade frame rate for resolution, enabling us to obtain an almost uniform high resolution across the scene (Fig. 3D, ii) after fusing data from 36 subframes in the periphery. For comparison, uniformly imaging the entire field of view at the higher resolution of the composite reconstruction (128 × 128 hr-pixels) would lower the global frame rate to 0.5 Hz. Therefore, in analogy to the resolution trade-off made in an individual subframe, using composite image formation with the linear constraint method, we can trade a higher frame rate in the center for a lower frame rate at the periphery.

***Space-variant PSF***

We note that, throughout the images presented here, the resolution is limited everywhere by pixel/cell sampling rather than by the diffraction limit of the optical system. Therefore, the space-variant PSF (SVPSF) in each subframe is defined by the exact configuration of the subframe’s cells; that is, the intensity from a single point in the scene is spread over the entire cell in which it is located in the image. For equivalently bright point sources, a point source found in a larger cell will yield a dimmer and more widely spread signal.

The SVPSF of both of the composite image reconstruction techniques that we demonstrate here is also inherited from the underlying cell grids of the subframes they are formed from. In the case of the weighted average method, the SVPSF is simply the locally weighted sum of the SVPSFs of each underlying subframe. Hence, the increase in resolution possible with this technique is limited (Fig. 3D, i). However, in the case of the linear constraint method, the inclusion of more subframes adds extra constraints to the set of simultaneous equations defining the image. This increases the number of independently recoverable cells in the image, thus reducing their average size and enhancing the SVPSF. This explains why the SVPSF of the linear constraint reconstruction tends to a uniformly sharp PSF (Fig. 3D, ii) when enough valid subframes are available.

The improvement in resolution with the linear constraint method comes at the expense of reconstruction speed. The weighted averaging technique is fast to compute [scaling as *O*(*N*)] and thus can easily be performed in real time at well above video rates for the resolutions presented here. In contrast, the linear constraint method involves finding the least-squares solution to a set of simultaneous equations [scaling as *O*(*N*^3^) in our method; see section S5]. Here, this reconstruction was carried out in postprocessing; however, the use of graphics processors and efficient matrix manipulation could potentially make this problem tractable in real time for the resolutions demonstrated here (*49*).

In the next section, we show how the data gathering capacity of our imaging system can be further improved by dynamically repositioning the fovea within the field of view in response to recent measurements and by accounting for parts of the scene that are moving in the reconstruction algorithms.

### Fovea gaze control

As we have described above, the position of the fovea in each subframe is determined by displaying a particular subset of the patterns that have been preloaded on the DMD. Therefore, mimicking the saccadic movement of animal vision, using real-time feedback control, the fovea can be rapidly repositioned in response to cues from previous images, for example, to follow motion of objects within the field of view or to move to areas anticipated to contain high levels of detail.

***Motion tracking***

A range of image analysis techniques exist to estimate motion in dynamic scenes, most involving some form of comparison between two consecutive frames (*50*, *51*). However, image comparison becomes more complicated if the pixel footprints change from one image to the next. Therefore, we have two competing requirements: Changing the locations of pixel boundaries is advantageous because it enables digital resolution enhancement of static parts of the scene (as demonstrated in Fig. 3C), yet determining which parts of the scene are in motion is easier if pixel boundaries remain constant between consecutive frames.

**Fig. 4:**
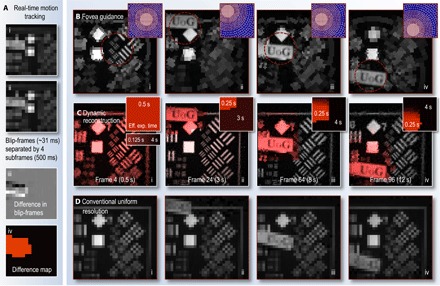
Fovea guidance by motion tracking. (**A**) Low-resolution blip-frames (i to ii), recorded after every fourth subframe. The difference between consecutive blip-frames reveals regions that have changed (iii). A binary difference map (iv) is then constructed from (iii) (see Materials and Methods for details). This analysis is performed in real time, enabling the fovea relocation to a region of the scene that has changed in the following subframes. (**B**) Frame excerpts from movie S3 showing examples of subframes (each recorded in 0.125 s) guided using blip-frame analysis to detect motion (fovea location updated at 2 Hz). The purple insets show the space-variant cell grid of each subframe. (**C**) Frame excerpts from movie S4 showing the reconstructed (using linear constraints) video stream of the scene also capture the static parts of the scene at higher resolution. Here, difference map stacks (shown as insets) have been used to estimate how recently different regions of the scene have changed, guiding how many subframes can contribute data to different parts of the reconstruction. This represents an effective exposure time that varies across the field of view. Here, the maximum exposure time has been set to 4 s (that is, all data in the reconstruction are refreshed at most after 4 s), and the effective exposure time has also been color-coded into the red plane of the reconstructed images. (**D**) Conventional uniform-resolution computational images of a similar scene for comparison (also shown in movie S4). These use the same measurement resource as (B) and (C). Section S7 gives a detailed description of movies S3 and S4.

To balance these requirements, we vary the cell boundaries of consecutive space-variant resolution subframes, as described above, but also interlace these frames with a short-exposure frame of uniform low resolution (for clarity, henceforth referred to as a blip-frame). The pixel boundaries of the blip-frames never change, and we use comparison of consecutive blip-frames to detect scene motion, as shown in Fig. 4A. We select relative resolutions for the subframes (1024 cells) and blip-frames (16 × 16 uniform pixels) and the interlacing frequency (2 Hz) to minimize the impact of the blip-frames on the overall frame rate. In the examples here, interlacing with a blip-frame reduces the average frame rate by only ~7%. Alternatively, to avoid the use of blip-frames, we could reconstruct pairs of subframes with identical pixel footprints and look for changes between these to track motion. However, this strategy would reduce the supersampling rate by a factor of 2.

Movie S3 and Fig. 4B show how motion tracking is used to guide the position of the fovea in real time (see section S7 for full description of movie S3). Here, the fovea follows a moving sign containing detail in the form of the letters “UoG,” as it is manually swept in front of the camera. The subframe frame rate is 8 Hz, and in between every blip-frame, we incorporate a “fixation phase,” where the fovea stays in the same area but performs four subframe digital supersampling, as described above. This strategy captures enough information at that location to double the linear resolution within the fovea should the scene remain static. We also inject a stochastic element into the fovea movement: For a randomly chosen fraction *p* of the subframes, the fovea is positioned in a random location not recently accessed, where, here, *p* ~ 20%. This ensures that all of the subframes are at least intermittently sampled, improving the quality of the longer exposure reconstruction of static parts of the scene.

In addition to guiding the location of the fovea to fast-changing parts of the scene, the blip-frames also enable the construction of a dynamic map, estimating how recently different regions of the scene last changed. Composite higher-resolution frames can then be reconstructed using stacks of difference maps to determine the local effective exposure time across the field of view (that is, how many earlier recorded subframes can contribute data to each region of the reconstruction). Using scene motion estimation to drive both the fovea movement and to build composite images results in a dynamic video reconstruction, which can have significantly enhanced detail in comparison with conventional uniform-resolution imaging. This is demonstrated in movie S4 and by comparing Fig. 4C to Fig. 4D (see section S7 for full description of movie S4). Figure 4C shows examples of composite frame reconstructions using foveated imaging and difference map stacks. The local effective exposure time has been color-coded into the red channel of the image, highlighting how it changes as the scene evolves. Examples of the stacked difference maps (which also represent the effective exposure time of frames in the reconstruction) are shown as insets. Figure 4D shows conventional uniform-resolution computational images of a similar scene under the same measurement resource. Here, all image data are refreshed at a frame rate of 8 Hz; however, unlike Fig. 4C, the resolution is never high enough to capture detail of the lettering or of the higher-resolution parts of the calibration grids.

***Detail estimation***

Depending on the nature of a dynamic scene, the entire field of view may sometimes be temporarily static over the course of several subframes. However, beginning to record a uniform high-resolution image at this point is not necessarily the optimum strategy: Such a measurement may be quickly interrupted because we have no knowledge of how long the scene will remain static. Therefore, it is prudent to attempt to measure the most detail-rich parts of the image first.

**Fig. 5:**
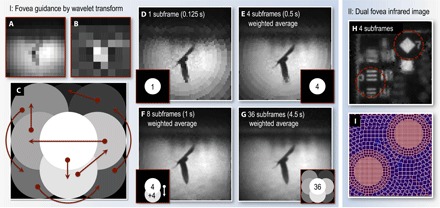
Detail estimation and infrared dual fovea reconstruction. (**A** to **C**) Fovea guidance by wavelet transform. (A) The fovea trajectory is determined by first measuring a blip-frame. A single-tier Haar wavelet transform is then performed on (A) to produce an edge contrast map (B) from which the fovea trajectory is then determined (for details, see Materials and Methods). (C) Map of the fovea trajectory within the field of view. Brighter regions indicate areas that the fovea visits earlier. Arrows show the trajectory of the fovea. (**D** to **G**) Image reconstructions after sampling the scene with various numbers of subframes and fovea positions. In this example, the fovea trajectory determined by the wavelet transform samples most of the detail in the scene after eight subframes. This is 50% of the time required to sample the entire field of view at the same resolution as the center has been sampled here. (**H** and **I**) Dual fovea infrared image. (H) Weighted average of four subframes (1368 cells per subframe; frame rate, 6 Hz), each having two fovea. (I) Cell grid of one of the subframes.

In our system, we aim to achieve this by performing a single-tier Haar wavelet transform on the blip-frame, which yields information about the location of edges in the image and hence regions of fine detail [see Materials and Methods; Aβmann and Bayer (*21*) and Burrus *et al*. (*52*) provide a detailed description of the Haar transform]. We use this to calculate a fovea trajectory that samples regions weighing most heavily in the wavelet transformation first, as shown in Fig. 5.

***Manual control***

Complementing the automated fovea guidance techniques described above, we have also implemented a manual fovea control system, where a user can click on the part of the scene they wish to view in high resolution. Other forms of manual control could also be envisaged. For example, control by a single operator could be implemented by measuring eye movements using a gaze tracker and by placing the high-resolution fovea wherever the operator looked. In this case, by scaling the resolution profile of the patterns to match the radial visual acuity profile of the eye, the scene could appear to the user to be rendered practically seamlessly in uniformly high resolution (*35*).

## DISCUSSION AND CONCLUSIONS

Here, we have demonstrated that the data gathering capacity of a single-pixel computational imaging system can be enhanced by mimicking the adaptive foveated vision that is widespread in the animal kingdom. Unlike a simple zoom, in our system every frame delivers new spatial information from across the entire field of view, and so, this framework rapidly records the detail of fast-changing features while simultaneously accumulating enhanced detail of more slowly changing regions over several consecutive frames. This tiered supersampling approach enables the reconstruction of video streams where both the resolution and the effective exposure time vary spatially and adapt dynamically in response to the evolution of the scene.

Unlike many compressive sensing algorithms, our foveated imaging strategy does not require a priori knowledge of the basis in which the image can be sparsely represented (*16*). Instead, we rely on the assumption that only some regions within the field of view will change from frame to frame. For many dynamic scenes, this is a reasonable assumption and one that animal vision systems have evolved to incorporate. Our foveated imaging system could potentially be further enhanced if used in conjunction with compressive sensing algorithms, at both the sampling stage (by concentrating measurements in an undersampled set toward the most important regions of the scene) and reconstruction stage [by incorporating any additional a priori knowledge of the scene to improve accuracy and reduce noise in the composite images (*53*)]. Section S6 describes this possibility in more detail. We also note that our composite reconstruction technique is similar in concept to the strategy used in some forms of video compression, which also rely on estimation of how recently local regions of the scene have changed (*54*, *55*).

We have demonstrated our system at visible wavelengths; however, the technique is, of course, not limited to the visible. For example, Fig. 5H (i) shows a short-wave infrared (SWIR) image recorded in the wavelength range of 800 to 1800 nm through a piece of Perspex that is opaque to visible light (*44*). This is realized by exchanging the APD with a SWIR-sensitive diode and illuminating with a heat lamp. In addition, Fig. 5H (i) also has two fovea, which highlights that the number of independently operating fovea can be increased should the scene demand it (*24*) and that these systems could also accommodate a range of additional visual adaptations both present in and beyond those found in the animal kingdom (*56*).

In what types of imaging systems might these approaches be most beneficial in the future? The techniques described here may be applied to any form of computational imager performing reconstructions from a set of sequentially made correlation measurements. Despite the challenges of low frame rates (or low SNR for equivalent frame rates) exhibited by single-pixel techniques in comparison with multipixel image sensors, there are a growing number of situations where conventional cameras cannot easily be used and single-pixel techniques prove highly desirable. For example, recently, it has been shown that a form of single-pixel imaging provides a powerful method to transmit image data of a fluorescent scene through precalibrated scattering media (such as diffusers or multimode fibers) (*13*–*15*). Single-pixel techniques also make it possible to image at wavelengths where single-pixel detectors are available, but multipixel image sensors are not (*5*–*7*). In all of these systems, there is a trade-off between resolution and frame rate, and our work demonstrates a flexible means to adaptively optimize this trade-off to suit the nature of the dynamic scene under investigation.

Ultimately, beyond specific technical challenges, the performance of an adaptive foveated computational imaging system will be determined by the sophistication of the algorithms driving the way the scene is sampled. Here, we have demonstrated motion tracking using a relatively simplistic algorithm; however, the fields of machine and computer vision offer a wealth of more advanced approaches, such as motion flow algorithms, intelligent pattern recognition, and machine learning (*57*–*60*). The performance of future computational imaging systems can be enhanced by deploying the spatially variant sampling and reconstruction strategies we have demonstrated here, in partnership with sophisticated image analysis techniques designed to accommodate a variety of real-world situations.

## MATERIALS AND METHODS

### Experimental setup

The scene was imaged onto a DMD (Texas Instruments Discovery 7001 with ViALUX software) using a Nikon F-mount camera lens (AF NIKKOR 85mm 1:1:8D). The DMD operates as a dynamic mask: Light from a subset of the micromirrors is reflected to an APD (Thorlabs PMM02). The APD recorded the total intensity transmitted by each binary masking pattern. The scene was flood-illuminated with a light-emitting diode torch (Maglite).

### Correlation measurements in the stretched Hadamard basis

As described in the main text, we performed our measurements in a stretched Hadamard basis **s.** The elements of **s** took values of +1 or –1. However, our experimental implementation used a DMD that could represent masks that transmitted (mirrors “on”) or blocked (mirrors “off”) intensity regions within the image. This corresponded to masks consisting of +1 (transmitted light) and 0 (blocked light), but not the −1 required in **s.** This problem was circumvented by performing a double exposure for each measurement: first, displaying a “positive” pattern of +1s and 0s (in place of the −1s), yielding signal *b*_*n*_^pos^ for pattern *n*, followed by the “negative” of this pattern (that is, where the positions of 1s and 0s have been swapped), yielding signal *b*_*n*_^neg^. The weighting of the stretched Hadamard basis vector *b*_*n*_ can then be emulated by subtraction of the intensity transmitted by the negative pattern from the positive pattern; here, we used the normalized subtraction: *b*_*n*_ *=* (*b*_*n*_^pos^ − *b*_*n*_^neg^)/(*b*_*n*_^pos^ + *b*_*n*_^neg^). This double-exposure strategy also helped to cancel out any fluctuations in the scene illumination that occurred during the recording of a single subframe. More detail is given in sections S1 and S3.

### Real-time motion tracking

To track scene motion in real time, low-resolution blip-frames were recorded after every fourth subframe, as shown in Fig. 4A (i and ii). We note that the choice of blip-frame frequency will depend on the anticipated level of motion in the scene. The difference between consecutive blip-frames revealed regions that had changed (shown in Fig. 4A, iii). A binary difference map (example shown in Fig. 4A, iv) was then constructed by thresholding the modulus of Fig. 4A (iii) and then implementing a convex hull operation on the thresholded region to fill any gaps. Finally, a dilate operation expanded the highlighted area in every direction to ensure that it was large enough to accommodate the moving object. We then calculated the center of mass of the highlighted area and displayed sampling patterns that contained the fovea nearest to this coordinate. These operations were performed in real time, enabling fovea guidance in real time.

### Haar wavelet transform for identification of detail-rich parts of the field of view

When the scene was deemed static, we aimed to identify parts of the image that were likely to contain higher levels of detail. Once the uniform-resolution blip-frame was obtained (denoted here by 2D array **O**_blip_ of size *N*_blip_ × *N*_blip_ pixels), we first performed a single-tier Haar wavelet transform on it, yielding the 2D array **W** of the same size as **O**_blip_. Following Aβmann and Bayer (*21*), this process can be described as two consecutive operations. We first calculated the intermediate transform **W′**, which is given by *W*′(*i*,*j*) = *O*_blip_(2*i*,*j*) + *O*_blip_(2*i* + 1,*j*) and *W*′(*i* + *N*_blip_/2,*j*) = *O*_blip_(2*i*,*j*) − *O*_blip_(2*i +* 1,*j*) for *i* < *N*_blip_/2, where *i* and *j* index pixel coordinates in **W′.** These operations essentially encoded the sum and difference between adjacent rows of pixels in **O**_blip_. We then repeated this operation along the second dimension (that is, columns) to find **W**, that is, *W*(*i*,*j*) = *W*′(*i*,2*j*) + *W*′(*i*,2*j* + 1) and *W*(*i*,*j* + *N*_blip_/2) = *W*′(*i*,2*j*) − *W*′(*i*,2*j* + 1) for *j* < *N*_blip_/2. **W** consists of four quadrants: One is a coarse representation of the original image (scaled down by a factor of 2). The other three quadrants contained information about the contrast of horizontal, vertical, and diagonal edges present in **O**_blip_ on the scale of the **O**_blip_ pixels. Regions containing high contrast on the scale of the blip-frame pixels returned high values in the wavelet transform. We combined the edge contrast data into a single contrast map (example shown in Fig. 5B) by calculating the quadratic sum of the three quadrants containing edge information, which was then used to determine the fovea trajectory. This was achieved by ordering the preloaded fovea patterns according to which fovea are nearest the regions of high edge contrast. In particular, all fovea were ordered by iteratively finding the nearest (thus far unused) fovea location to the region containing the highest (thus far unsampled) contrast. An example of a fovea trajectory map is shown in Fig. 5C.

### Dual fovea infrared image

For this infrared experiment, the camera setup was slightly modified from the description above: The APD was replaced with an InGaAs detector (Thorlabs PDA20CS InGaAs; 800 to 1800 nm), and the scene was illuminated with a heat lamp.

## Supplementary Material

http://advances.sciencemag.org/cgi/content/full/3/4/e1601782/DC1

## SUPPLEMENTARY MATERIALS

Supplementary material for this article is available at http://advances.sciencemag.org/cgi/content/full/3/4/e1601782/DC1 section S1. Hadamard correlation measurements section S2. Foveated subframe reconstruction section S3. Signal-to-noise ratio section S4. Weighted averaging image fusion section S5. Linear constraint image fusion section S6. Reconstructions with additional assumptions section S7. Supplementary movie file descriptions fig. S1. Reconstruction comparison. fig. S2. Movie S1 snapshot. fig. S3. Movie S2 snapshot. fig. S4. Movie S3 snapshot. fig. S5. Movie S4 snapshot. movie S1. Real-time subframe display. movie S2. Postprocessed linear constraint reconstruction. movie S3. Real-time motion tracking and fovea guidance. movie S4. Real-time weighted averaging and postprocessed linear constraint reconstruction of a dynamic scene. References (*61*–*63*)
