# Supplementary material for: Adaptive foveated single-pixel imaging with dynamic supersampling
Source: Sci Adv. 2017 Apr 21;3(4):e1601782. doi: 10.1126/sciadv.1601782 (PMC5400451; doi:10.1126/sciadv.1601782)
Supplement: http://advances.sciencemag.org/cgi/content/full/3/4/e1601782/DC1 [file supp_3_4_e1601782__index.html]

Science Advances | Science Advances

## Supplementary Materials

**This PDF file includes:**

- section S1. Hadamard correlation measurements
- section S2. Foveated subframe reconstruction
- section S3. Signal-to-noise ratio
- section S4. Weighted averaging image fusion
- section S5. Linear constraint image fusion
- section S6. Reconstructions with additional assumptions
- section S7. Supplementary movie file descriptions
- fig. S1. Reconstruction comparison.
- fig. S2. Movie S1 snapshot.
- fig. S3. Movie S2 snapshot.
- fig. S4. Movie S3 snapshot.
- fig. S5. Movie S4 snapshot.
- Legends for movies S1 to S4
- References (*61–63*)

Download PDF

**Other Supplementary Material for this manuscript includes the following:**

- movie S1 (.mov format). Real-time subframe display.
- movie S2 (.mov format). Postprocessed linear constraint reconstruction.
- movie S3 (.mov format). Real-time motion tracking and fovea guidance.
- movie S4 (.mov format). Real-time weighted averaging and postprocessed linear constraint reconstruction of a dynamic scene.

**Files in this Data Supplement:**

- Adobe PDF - 1601782\_SM.pdf
