# Supplementary material for: Adaptive foveated single-pixel imaging with dynamic supersampling
Source: Sci Adv. 2017 Apr 21;3(4):e1601782. doi: 10.1126/sciadv.1601782 (PMC5400451; doi:10.1126/sciadv.1601782)
Supplement: http://advances.sciencemag.org/cgi/content/full/3/4/e1601782/DC1 [file 1601782_SM.pdf]

Science Advances

AAAS

advances.sciencemag.org/cgi/content/full/3/4/1601782/DC1

Supplementary Materials for

Adaptive foveated single-pixel imaging with dynamic supersampling

David B. Phillips, Ming-Jie Sun, Jonathan M. Taylor, Matthew P. Edgar, Stephen M. Barnett, Graham M. Gibson, Miles J. Padgett

Published 21 April 2017, *Sci. Adv.* **3**, e1601782 (2017)
DOI: 10.1126/sciadv.1601782

The PDF file includes:

- section S1. Hadamard correlation measurements
- section S2. Foveated subframe reconstruction
- section S3. Signal-to-noise ratio
- section S4. Weighted averaging image fusion
- section S5. Linear constraint image fusion
- section S6. Reconstructions with additional assumptions
- section S7. Supplementary movie file descriptions
- fig. S1. Reconstruction comparison.
- fig. S2. Movie S1 snapshot.
- fig. S3. Movie S2 snapshot.
- fig. S4. Movie S3 snapshot.
- fig. S5. Movie S4 snapshot.
- Legends for movies S1 to S4
- References (*61*–*63*)

Other Supplementary Material for this manuscript includes the following:
(available at advances.sciencemag.org/cgi/content/full/3/4/e1601782/DC1)

- movie S1 (.mov format). Real-time subframe display.
- movie S2 (.mov format). Postprocessed linear constraint reconstruction.
- movie S3 (.mov format). Real-time motion tracking and fovea guidance.
- movie S4 (.mov format). Real-time weighted averaging and postprocessed linear constraint reconstruction of a dynamic scene.

section S1: Hadamard correlation measurements

A Hadamard matrix is defined as an $N\times N$ matrix with elements that take the values of +1 or -1, and with rows that are orthogonal to one another. The supplementary information of reference [7], and the references therein give an excellent description of the generation and use of the Hadamard matrices.

In summary, a 2D uniform resolution mask of index n is formed by reformatting row n of the Hadamard matrix into a uniform 2D grid, as shown in Figs. 2(a-b). However, our experimental implementation uses a DMD that can represent masks that transmit (mirrors 'on') or block (mirrors 'off') intensity regions within the image. This corresponds to masks consisting of +1 (transmitted light) and 0 (blocked light), but not the -1 required by the Hadamard matrix. This problem is circumvented by performing a double exposure for each measurement: first displaying a 'positive' pattern of +1s and 0s (in place of the -1s), yielding signal a_n^{pos} for Hadamard pattern n, followed by the 'negative' of this pattern (i.e. where the positions of 1s and 0s have been swapped), yielding signal a_n^{neg}. The weighting of the Hadamard basis vector, a_n, can then be emulated by subtraction of the intensity transmitted by the negative pattern from the positive pattern, here we use the normalised subtraction:

$$a_n = \frac{a_n^{pos} - a_n^{neg}}{a_n^{pos} + a_n^{neg}}. \tag{1}$$

Although halving the frame-rate, this double exposure strategy also acts to cancel out some of the noise due to fluctuations (at frequencies lower than 10kHz) in the ambient illumination (see S3 below), a technique analogous to differential ghost imaging [61].

section S2: Foveated sub-frames reconstruction

Here we derive and discuss in further detail Equation 2 of the main text. We will make use of the transformation matrix $\boldsymbol{T}$, which maps from N-element cell space to the larger M-element hr-pixel space. $\boldsymbol{T}$ is an $M\times N$ binary matrix where the locations of the 'ones' in column n denote the hr-pixels that belong to cell n.

With the help of this matrix, we can define a new basis $\boldsymbol{s}_n$, formed by "stretching" the Hadamard vectors $\boldsymbol{h}_n$ to conform to our nonuniform pixel grid:

$$\boldsymbol{s}_n = \boldsymbol{T}\boldsymbol{h}_n, \tag{2}$$

These are the raw patterns that we will measure using our DMD. It is important to note that, in contrast to conventional computational imaging using Hadamard matrices on a regular grid, the vectors $\boldsymbol{s}_n$ are not orthogonal in hr-pixel space. However, after some matrix algebra it can be shown that there exists a dual basis $\tilde{\boldsymbol{s}}_m = \boldsymbol{A}^{-1}\boldsymbol{s}_m$ forming a biorthogonal set with $\boldsymbol{s}_n$, i.e. the following relation holds:

$$\boldsymbol{s}_n^T\tilde{\boldsymbol{s}}_m = \boldsymbol{s}_n^T\boldsymbol{A}^{-1}\boldsymbol{s}_m = N\delta_{mn}.$$

Here $\boldsymbol{A}$ is an $M\times M$ diagonal matrix such that A_{mm} is equal to the area of the cell to which hr-pixel m belongs.

The existence of this biorthogonality relationship makes it helpful to represent our high-resolution object $\boldsymbol{o}$ in the dual basis $\tilde{\boldsymbol{s}}_m$ as follows:

$$\boldsymbol{o} = \frac{1}{N}\sum_{m=1}^{N} b_m\,\boldsymbol{A}^{-1}\,\boldsymbol{s}_m + \boldsymbol{\epsilon}, \tag{3}$$

where $\boldsymbol{\epsilon}$ represents those high-spatial-frequency components that are orthogonal to all $\boldsymbol{s}_n$ (i.e. $\boldsymbol{s}_n^T\boldsymbol{\epsilon} = 0$ for all n), and hence that the imaging system described here is not sensitive to.

Then, by projecting $\boldsymbol{o}$ onto our basis set $\boldsymbol{s}_n$ and expanding, we can show that $\boldsymbol{s}_n^T\,\boldsymbol{o} = b_n$ as follows:

$$\begin{aligned}\boldsymbol{s}_n^T\,\boldsymbol{o} &= \boldsymbol{s}_n^T\frac{1}{N}\sum_{m=1}^{N} b_m\,\boldsymbol{A}^{-1}\,\boldsymbol{s}_m + \boldsymbol{s}_n^T\boldsymbol{\epsilon}\\ &= \frac{1}{N}\sum_{m=1}^{N} b_m\,\boldsymbol{s}_n^T\,\boldsymbol{A}^{-1}\,\boldsymbol{s}_m = b_n.\end{aligned} \tag{4}$$

These measurements can then be used to derive our estimate $\boldsymbol{o}_{sv}$ of the object (Equation 2 in the main text):

$$\boldsymbol{o}_{sv} = \boldsymbol{A}^{-1}\frac{1}{N}\sum_{n=1}^{N} b_n\boldsymbol{s}_n. \tag{5}$$

However, if we substitute (2) into this equation then we see that the same reconstruction in fact be computed more efficiently in cell space:

$$\boldsymbol{o}_{sv} = \frac{1}{N}\sum_{n=1}^{N} b_n \boldsymbol{A}^{-1}\boldsymbol{T}\boldsymbol{h}_n = \boldsymbol{A}^{-1}\boldsymbol{T}\frac{1}{N}\sum_{n=1}^{N} b_n \boldsymbol{h}_n. \tag{6}$$

Although this matrix equation is slightly more long-winded than (2), it shows that the reconstruction can be performed in the (lower-dimensional) cell space, and then the final result remapped just once onto the uniform hr-pixel grid.

section S3: Signal-to-noise ratio

There are a variety of different ways signal-to-noise ratio (SNR) can be defined for images. For a uniform resolution image, here we define SNR as:

$$\text{SNR} = \frac{\sigma_{\boldsymbol{o}}}{<\boldsymbol{\sigma}>}, \tag{7}$$

where $\sigma_{\boldsymbol{o}}$ is the standard deviation of pixel intensities in a noiseless ideal image $\boldsymbol{o}$ (which may be approximated by averaging a large number of sequentially recorded images of an unchanging scene). Therefore $\sigma_{\boldsymbol{o}}$ provides a measure of the level of signal variation we expect to measure in the image. $\boldsymbol{\sigma}$ is a vector of M elements representing the standard deviation of the fluctuation due to noise in each pixel, measured from the same large number of images. $<\boldsymbol{\sigma}>$ denotes the mean value of the elements in $\boldsymbol{\sigma}$, i.e. the standard deviation of the noise fluctuations in each pixel, averaged over all of the pixels in the image. In our experiments SNR depends heavily on the illumination conditions. A typical example for a 64×64 pixel image recorded using the double exposure technique (see S1) under similar conditions to our experiments in this work, is an SNR of ~ 10 (measured using 140 frames).

There are two main source of noise in our experiments: (i) noise in the photodiode measurement, and (ii) fluctuations in illumination intensity during the measurement of a single image. Using Hadamard patterns rather than, for example, raster scanning a single pixel across the DMD, raises the level of illumination reaching the photodiode for each pattern, and so serves to combat (i). Performing the double exposure technique serves to combat (ii) by going some way towards normalising intensity fluctuations that occur on a timescale slower than 10 kHz (half of the DMD modulation rate). For example, performing only a single exposure per pattern measurement (e.g. just positive patterns), significantly degrades the SNR in the above example from SNR ~ 10 to ~ 0.5.

In passive uniform resolution single-pixel imaging techniques, the SNR scales approximately in inverse proportion to the square of the linear resolution, for a given constant exposure-time. Following from this observation, we expect the SNR in our spatially-variant resolution imaging system to vary across each individual sub-frame, with the higher resolution regions being most sensitive to noise. Therefore the local SNR scales in inverse proportion to the square of the local linear resolution, which is what we observed qualitatively during our experiments. Therefore a single number can no longer meaningfully be used to capture the SNR of the image. The weighted-averaging method does go some way towards improving the SNR, as is discussed in more detail in [45].

However, our space-variant imaging system does offer a reduction in the noise caused by motion blur. When a scene changes *during* the measurement of a computational image using Hadamard patterns (uniform or spatially-variant), the reconstruction not only exhibits conventional motion blur, but also a splash of noisy pixels across the field-of-view. This *pattern multiplexing* noise is due to scene movement causing inconsistencies in the measured weights of each pattern. By lowering the resolution in regions of the scene deemed static, our foveated imaging system reduces the amount of time required to image a moving part of the scene to a given resolution (in the examples here, by a factor of 4), therefore reducing both conventional motion blur and pattern multiplexing noise.

section S4: Weighted-averaging image fusion

Each hr-pixel is formed by weighting the contribution of data from each sub-frame in inverse proportion to the area of the corresponding sub-frame cell that the data is taken from. Therefore the intensity of pixel i, j in the weighted mean composite image is given by:

$$O_{wm}(i,j) = \frac{1}{B(i,j)}\sum_{k=1}^{K}\frac{O^{(k)}(i,j)}{A^{(k)}(i,j)}, \tag{8}$$

where k indexes the sub-frames used to calculate the composite image and K is the total number of sub-frames to combine. $o^{(k)}(i,j)$ is the value of pixel i, j in space-variant sub-frame k, and $A^{(k)}(i,j)$ is the area of the cell

that pixel i, j belongs to. $B(i,j) = \sum_{k=1}^{K}(A^{(k)})^{-1}(i,j)$ serves to normalise the sum. Consistent with our earlier vector notation, we can equivalently write:

$$\boldsymbol{o}_{wm} = \boldsymbol{B}^{-1}\sum_{k=1}^{K}(\boldsymbol{A}^{(k)})^{-1}\,\boldsymbol{o}^{(k)}, \tag{9}$$

where $\boldsymbol{B} = \sum_{k=1}^{K}(\boldsymbol{A}^{(k)})^{-1}$. $O_{wm}(i,j)$ is element i, j of the 2D image denoted by matrix $\boldsymbol{O}_{wm}$, where $\boldsymbol{O}_{wm} =$ reshape$[\boldsymbol{o}_{wm}, m_p, m_p]$, and $m_p = M^{\frac{1}{2}}$, the number of hr-pixels along one side of the square image. Equations 8 and 9 therefore specify an equal weighting of sub-frames within the fovea (where the pixels are all of the same size), and in the peripheral region promotes data from pixels that have a smaller area and thus a higher local resolution. This strategy incorporates local data from all sub-frames in every composite image pixel, which has the benefit of suppressing noise.

We note that a variety of other weightings may also be applied. Other examples that we investigated include using only data from the sub-frame with the highest resolution pixel (with equal weighting given in regions where sub-frames have the same sized pixels), and the weighting of more recent measurements more prominently. This second weighting strategy can be applied if some parts of the scene are expected to change throughout the measurement. The weighting choice depends upon the distribution of pixel areas in the sub-frames, the noise levels in the measurement, and the expected level of scene motion.

section S5: Linear-constraints image fusion

For our linear-constraint algorithm, we fuse information from multiple sub-frames by forming a system of linear equations representing constraints on the high-resolution reconstructed image. The problem can be expressed as the matrix equation:

$$\mathbb{T}\boldsymbol{o}'_{sv} = \mathcal{C}, \tag{10}$$

where $\mathbb{T}$ and $\mathcal{C}$ are composed of information from multiple sub-frames as follows:

$$\begin{pmatrix} (\boldsymbol{T}^{(k=1)})^T \\ (\boldsymbol{T}^{(k=2)})^T \\ \vdots \\ (\boldsymbol{T}^{(k=K)})^T \end{pmatrix} \boldsymbol{o}'_{sv} = \begin{pmatrix} \boldsymbol{c}^{(k=1)} \\ \boldsymbol{c}^{(k=2)} \\ \vdots \\ \boldsymbol{c}^{(k=K)} \end{pmatrix}. \tag{11}$$

Here $\boldsymbol{o}'_{sv}$ is our improved estimate of the original scene fusing data from multiple sub-frames. $\boldsymbol{T}^{(k)}$ is our binary stretching transform matrix as defined as above. As before, k indexes the sub-frames used to calculate the composite image, and K is the total number of sub-frames combined. Therefore here $(\boldsymbol{T}^{(k)})^T$ is an $M \times N$ binary matrix encoding which hr-pixels belong to each cell (i.e. element m, n is 1 if pixel m belongs to cell n, and 0 otherwise). $\boldsymbol{c}^{(k)}$ is a column vector of length N, element n of which represents the sum of all the hr-pixel values in cell n of sub-frame k. Conveniently, the vector $\boldsymbol{c}$ is already computed as part of the reconstruction of sub-frame k (referring to Equation 6, we see that $\boldsymbol{c} = \frac{1}{N}\sum_{n=1}^{N} b_n \boldsymbol{h}_n$).

Note that in Eqs. 10 and 11, each individual constraint (i.e. row) only applies to a *localized* region of the composite image (i.e. one cell). This offers a natural method to account for local motion within the scene during the acquisition of the k sub-frames. Information about which parts of the scene have changed is encoded in the difference map stacks, and if motion is detected in a given cell then we can simply omit the constraints that apply to this position, for this and earlier times. This is achieved by deleting any affected rows from the matrices $\boldsymbol{T}^{(k)}$, along with the corresponding elements from vectors $\boldsymbol{c}^{(k)}$.

We also note that, in the same way as $\boldsymbol{T}$ maps from cell space to hr-pixel space, $\boldsymbol{T}^T$ maps from hr-pixel space to cell space. These transformations are related by: $\boldsymbol{T}^T\boldsymbol{A}^{-1}\boldsymbol{T} = \mathbf{1}$, indicating that conversion from cell to hr-pixel space and back again is lossless. However, the reverse transformation $\boldsymbol{A}^{-1}\boldsymbol{T}\boldsymbol{T}^T$ does not equal $\mathbf{1}$, indicating that a transformation from hr-pixel to cell space and back again is *not* lossless, and high resolution detail is lost in the transformation.

In practice, we solve for $\boldsymbol{o}'_{sv}$ using a least-squares method that is suitable for systems that may be locally overdetermined, critically determined, or underdetermined depending on the number of sub-frames available for the reconstruction. Specifically, in this work we used the LabVIEW *solve linear equations* function, although other routines in other languages may be more efficient. This function decomposes $\mathbb{T}$ into an orthogonal matrix $\boldsymbol{Q}$ and an upper triangular matrix $\boldsymbol{R}$, so that $\mathbb{T} = \boldsymbol{Q}\boldsymbol{R}$. Substituting for $\mathbb{T}$ in Eq. 10 yields $\boldsymbol{Q}\boldsymbol{R}\boldsymbol{o}'_{sv} = \mathcal{C}$, which can be rearranged to $\boldsymbol{R}\boldsymbol{o}'_{sv} = \boldsymbol{Q}^T\mathcal{C}$. This triangular system of equations is then solvable using recursive techniques.

Our linear-constraint method can be sensitive to noise in the sub-frame measurements, and in particular noise is amplified in the highest spatial frequencies of the composite image (i.e. within the fovea). If necessary we suppress this noise by applying a spatially-variant smoothing constraint to the system of equations, which is derived from the weighted average composite image formed from the raw data (which although has lower resolution, crucially also possess a lower level of noise - see ref [45]). This is achieved adding extra rows to the system of equations incorporating the information present in the weighted average composite image $\boldsymbol{o}_{wm}$, and solving the following matrix equation which enables us to weight the relative importance of the smoothing constraint using a weighting matrix $\boldsymbol{W}$:

$$\left[\mathbb{T}'^{T} \boldsymbol{W} \mathbb{T}'\right] \boldsymbol{o}''_{sv} = \left[\mathbb{T}'^{T} \boldsymbol{W}\right] \mathcal{C}', \tag{12}$$

$$\text{where:} \quad \mathbb{T}' = \begin{bmatrix} \mathbb{T} \\ \boldsymbol{I} \end{bmatrix}, \quad \mathcal{C}' = \begin{bmatrix} \mathcal{C} \\ \boldsymbol{o}_{wm} \end{bmatrix}, \tag{13}$$

Here $\boldsymbol{o}''_{sv}$ is the improved estimate of the original scene, now also including the noise suppression smoothing constraint. $\boldsymbol{I}$ is an $M \times M$ element identity matrix, with each row representing one hr-pixel. $\boldsymbol{I}$ therefore represents a system of equations expressing the weighted mean reconstruction $\boldsymbol{o}_{wm}$ in the hr-pixel basis. $\boldsymbol{W}$ is a $P \times P$ diagonal matrix, where P is the total number of rows of $\mathbb{T}'$. The value of the diagonal elements in the first D rows set to 1, where D is the total number of rows of $\mathbb{T}$. The diagonal elements in the remaining rows of $\boldsymbol{W}$ are set to the value w, which represents the weighting of the smoothing constraint terms in the reconstruction. This effectively gives us a tunable compromise between noise suppression and faithful reproduction of the high spatial frequencies close to the cut-off frequency of the reconstruction: for example a greater weighting of the constraint w leads to lower noise images but with high frequencies suppressed (non-uniformly across the field of view, reflecting the underlying measurements). In practical terms it is highly attractive to use the weighted average image as a constraint for the linear-constraint reconstruction, as it represents a ready-made space-variant noise suppression function which is near to the optimum answer, which would be non-trivial to otherwise synthesise from our irregular grids of sub-frame cells.

In our reconstructions, typically we used $1 \lesssim w \lesssim 10$. When $w = 0$, the reconstruction $\boldsymbol{o}''_{sv} = \boldsymbol{o}'_{sv}$ and the smoothing constraint is ignored. As $w \to \infty$, then $\boldsymbol{o}''_{sv} \to \boldsymbol{o}_{wm}$ (in practice $\boldsymbol{o}''_{sv}$ becomes practically indistinguishable from $\boldsymbol{o}_{wm}$ for values of $w \gtrsim 1 \times 10^5$). Once $\boldsymbol{W}$ is incorporated, Eqn. 12 is solved in an identical manner to Eqn. 10.

We note that the matrices in the equations presented here are highly sparse, and an efficient implementation of the reconstruction code benefits significantly from exploiting this property. Note also that in the case of a regular square pixel grid (e.g. Fig. 2(a)) the algebraic problem is separable in the x and y dimensions. This reduces the formal computational complexity of the problem, thus making it possible to implement a realtime reconstruction. However, it would be significantly more challenging to construct an irregular pixel grid (e.g. Fig. 2(d)) that is separable in this way, while still meeting the other requirements for our geometry.

section S6: Reconstructions with additional assumptions

The foveated sampling strategy described in S2 results in the reconstruction of images possessing irregularly sized and shaped cells. Each cell is formed from a group of hr-pixels, which form the underlying uniform higher resolution Cartesian pixel grid of each image. We perform enough measurements so as to critically sample the total intensity of each cell - so therefore we know the sum of the intensities of the hr-pixels inside each of the cells, however our measurements provide no information about how this intensity may be distributed between the hr-pixels within each cell. Therefore, in the case of the reconstruction of an individual sub-frame, our reconstruction method (Eqn. 5) assigns the same value to each hr-pixel inside a particular cell: the *average* intensity of hr-pixels within that cell (by dividing the known total measured intensity by the number of hr-pixels in that cell - which is the function of the $\boldsymbol{A}^{-1}$ term in Eqns. 5 and 6). This approach mirrors the standard raw output of conventional uniform resolution camera sensors in that the intensity distribution within a pixel is represented as uniform as there is no information available to determine how it is distributed at finer scales.

Employing the weighted average and linear constraints image fusion methods described above in S4 and S5 increases the number of individual cells in the reconstruction - therefore these cells are smaller, and also become more irregular in shape (the new shapes depending upon the relative boundary positions of the cells in each fused sub-frame). Similarly to the reconstruction of an individual sub-frame, in both of the sub-frame fusion methods we assign the same value to each hr-pixel inside these new cells: once again the average intensity of hr-pixels within that cell.

However, following ideas from conventional uniform resolution compressed sensing, it is also possible to incorporate additional assumptions about the nature of the scene to attempt to redistribute the intensity between

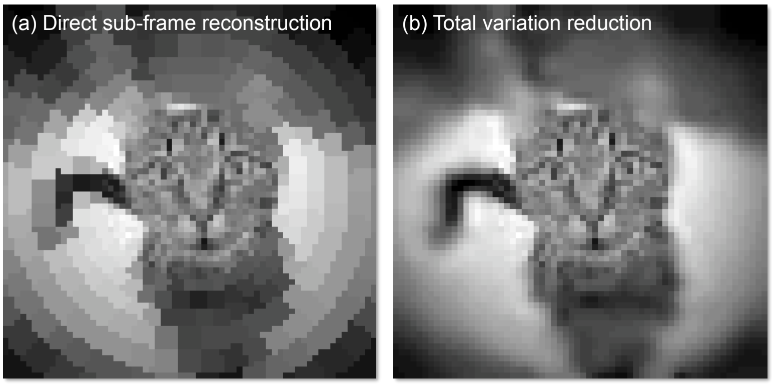

S1: **Reconstruction comparison**

hr-pixels within each cell in a more realistic manner. As our measurements for an *individual sub-frame* yield information only about low spatial frequencies in the periphery, we cannot hope recover any high spatial frequency detail in this region should it be present. However, under the assumption that the periphery has few high spatial frequencies, we can attempt to find a distribution of hr-pixels within each cell that yields a more smoothly varying intensity profile across the image.

Figure 1 shows a comparison of reconstructions of two 64×64 hr-pixel images from the same 1024 stretched Hadamard measurements. Figure 1(a) shows a direct fast reconstructive using Eqn. 6 as described in the main paper. This can be performed in real time at well above the 10 Hz frame-rate of our single-pixel camera. Figure 1(b) shows a reconstruction with the additional constraint of reducing the variation in intensity across the image, performed using the freely available CVX Matlab library [62,63]. We can see that the central critically sampled hr-pixels within the fovea remain unchanged, while the values of the hr-pixels in the peripheral cells have been adjusted to smooth the image. In this case we defined the variation in the image ($V = v^{\frac{1}{2}}$) as the square-root of sum of the squared absolute difference in hr-pixel values along the x and y dimensions, where:

$$v = \sum_{j=1}^{m_p} \sum_{i=1}^{m_p - 1} \left[|O'''_{sv}(i,j) - O'''_{sv}(i+1,j)|^2 \right] + \sum_{i=1}^{m_p} \sum_{j=1}^{m_p - 1} \left[|O'''_{sv}(i,j) - O'''_{sv}(i,j+1)|^2 \right], \tag{14}$$

where $O'''_{sv}(i,j)$ is element i,j of the 2D image denoted by matrix $\boldsymbol{O}'''_{sv}$, where $\boldsymbol{O}'''_{sv} = \mathrm{reshape}[\boldsymbol{o}'''_{sv}, m_p, m_p]$, $m_p = M^{\frac{1}{2}}$ as above, and $\boldsymbol{o}'''_{sv}$ is a vector representing the current estimate of the image with the intensity variation constraint. We note that here we did not optimise the efficiency of the reconstruction, and there are a wide variety of different optimisation techniques and software available, some of which may potentially be more suited and efficient at solving our system of foveated measurements. The reconstruction of the composite images from the information present in multiple sub-frames could also be performed and potentially improved by applying additional assumptions about the nature of the scene, for example knowledge of the basis in which the image can be sparely represented. A study of which compressive sensing algorithms perform optimally with foveated measurements is currently the subject of ongoing work.

section S7: Media file descriptions

movie S1: Real-time sub-frame display. This movie shows data presented in Fig. 3(a). The left hand panel shows the sub-frames captured at 8 Hz (and processed and displayed in real-time). The super-sampling from one frame to the next can be seen both within the fovea where they repeat every 4 frames, and in the periphery where they repeat every 36 frames (the same as the length of the movie). The right hand panel shows the cell grid for each frame.

movie S2: Post-processed linear constraints reconstruction. This movie shows data presented in Fig. 3(c). The left hand panel shows the frame-by-frame linear constraints reconstruction. The high resolution appears to spread from the centre as in the periphery each new frame is fused with the existing data to improve the reconstruction. Right hand panel shows the effective exposure-time across the field-of-view. Initially the entire field-of-view has the same effective exposure-time as only a single frame has been recorded. In the centre only the most recent 4 sub-frames are used in the reconstruction (hence an effective exposure-time of 0.5 s). Surrounding this data from progressively more frames back is used in the reconstruction (thus increasing the effective exposure time).

movie S3: Real-time motion tracking and fovea guidance. This movie shows data presented in Fig. 4(b). The top-left panel shows the low-resolution blip-frame (recorded after every 4th sub-frame in ~ 31 ms). The bottom-left panel shows the difference between the 2 most recent consecutive blip-frames. The region of the moving object is clearly visible. The blip-frame and blip-difference frame are reconstructed, analysed and displayed in real-time. The middle panel shows the sub-frames captured at 8 Hz (and processed and displayed in real-time). Here the fovea is programmed to follows the moving part of the scene to image it at high resolution. Additionally, 20% of the time the fovea is programmed to jump to a random location within the field-of-view that was not recently accessed. This is performed to ensure that all of the sub-frames are at least intermittently sampled, improving the quality of the longer exposure super-sampled reconstruction of

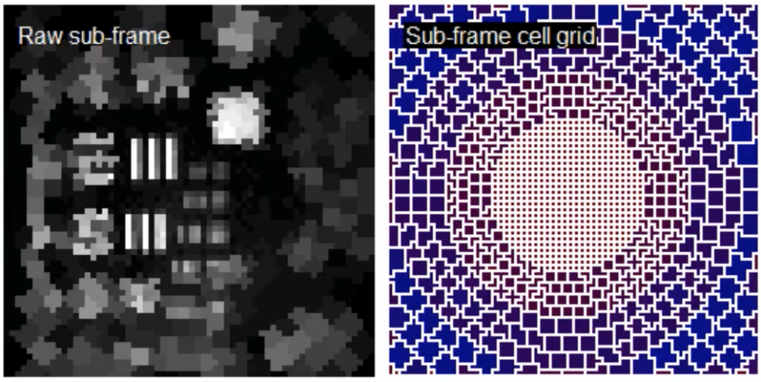

S2: **Media 1 snapshot**

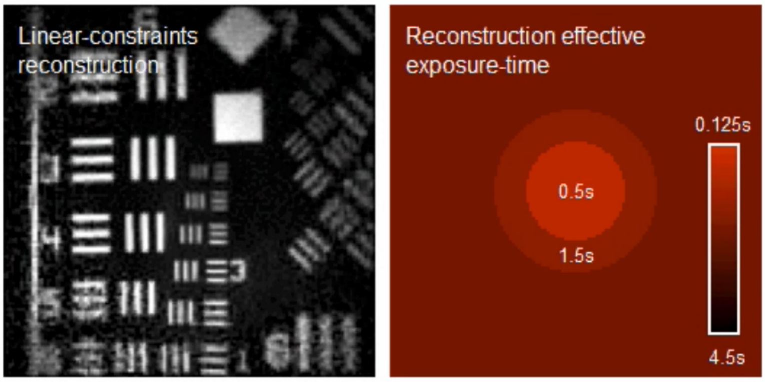

S3: **Media 2 snapshot**

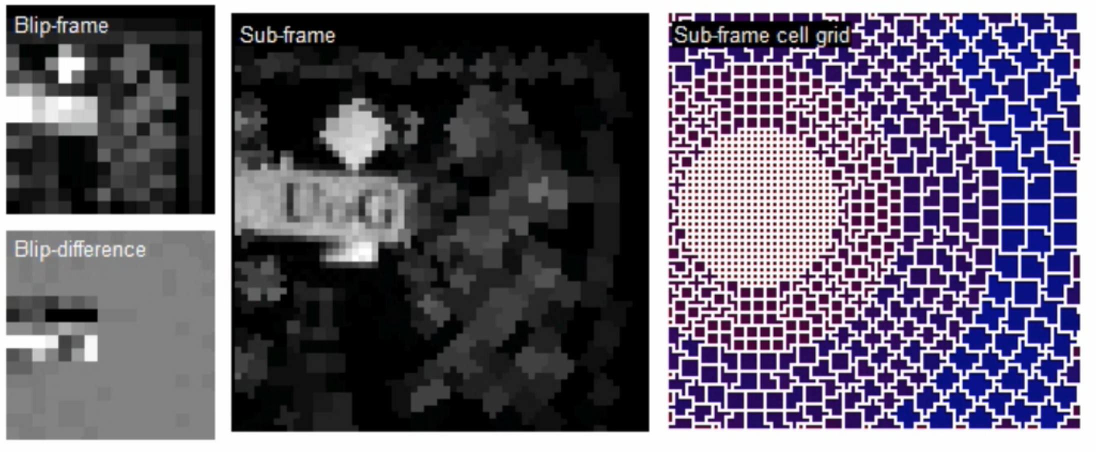

S4: **Media 3 snapshot**

static parts of the scene (shown in Media 4). The global position of the fovea is updated at 2 Hz (every 4 sub-frames), based on the blip-difference frame analysis. During each 4 sub-frame fixation phase in-between blip-frame measurements, the fovea cell footprints are shifted by 4 half-cell displacements in x and y [45]. This enables the resolution within the fovea to be doubled by combining the 4 measurements should the scene with the fovea remain static for the time of the measurements. The right hand panel shows the cell grid for each frame.

movie S4: Real-time weighted-averaging and post-processed linear constraints reconstruction of a dynamic scene. This movie shows data presented in Fig. 4(c-d). The top-left panel shows the post-processed linear-constraints reconstruction of the raw data shown in Media 3. The effective exposure-time of this reconstruction is shown in the top-right panel. Here the minimum effective exposure-time is 0.25 s and the maximum is 4 s. Therefore in the parts of the scene that are currently deemed to be moving, we display the

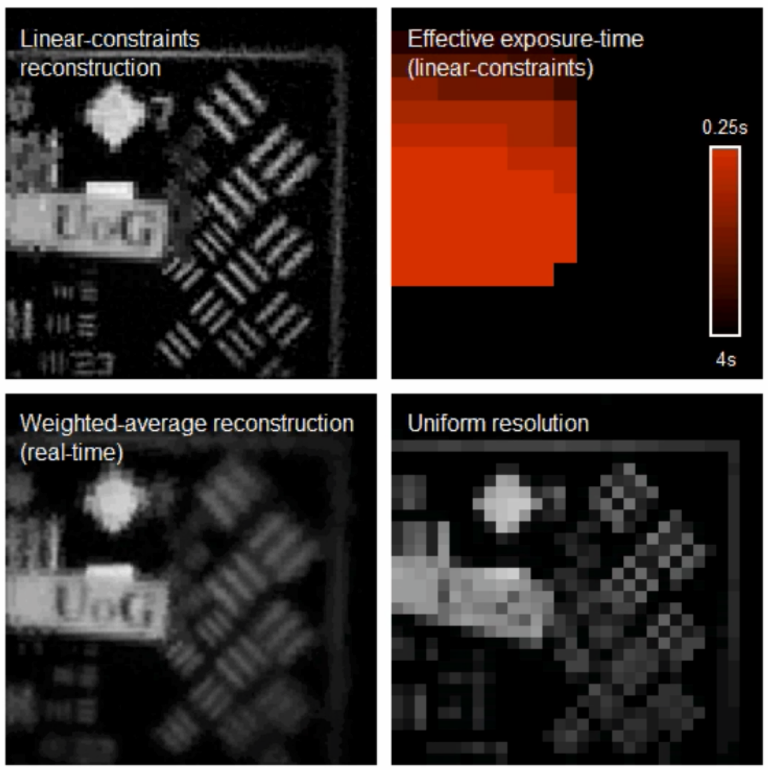

S5: **Media 4 snapshot**

average of the most recent 2 frames. In other regions the data used in this reconstruction is flushed after a maximum of 4 s (i.e. the maximum number of previous sub-frames from which data is used is 32). In this case as in some regions no change was detected throughout the entire duration of the clip (15 s, ~ 120 sub-frames), the maximum effective-exposure could have been 15 s. We show the case for a reduced 4 s maximum effective exposure-time to demonstrate the super-sampled recovery of the entire field-of-view. The bottom-left panel shows the real-time weighted-averaging reconstruction. Here instead of choosing a hard limit on the maximum effective exposure-time we have weighted older frames less prominently, which promotes recent measurements. The bottom-right panel shows a uniform resolution video of a similar scene, using the same measurement resource as the data in the other panels. Here the data is completely flushed every frame (0.125 s), however the resolution is never high enough to identify the lettering on the moving sign, or any of the features on the resolution target in the background.
